# What interventions affect the psychosocial burden experienced by prostate cancer patients undergoing active surveillance? A scoping review

**DOI:** 10.1007/s00520-022-06830-z

**Published:** 2022-01-26

**Authors:** Kim Donachie, Erik Cornel, Thomas Pelgrim, Leslie Michielsen, Bart Langenveld, Marian Adriaansen, Esther Bakker, Lilian Lechner

**Affiliations:** 1 HAN University of Applied Sciences, Academy of Health, P.O. Box 6960, 6503 GL Nijmegen, The Netherlands; 2 Department of Urology, ZGT Twente Hospital, Hengelo, The Netherlands; 3 HAN University of Applied Sciences, Nijmegen, The Netherlands; 4 Department of Cardiology, Zuyderland Medical Center, Sittard-Geleen, The Netherlands; 5 Faculty of Psychology, Open University, Heerlen, The Netherlands

**Keywords:** Active surveillance, Diet, Exercise, Lifestyle, Prostate cancer, Psychosocial support

## Abstract

**Purpose:**

Living with untreated prostate cancer (PCa) may cause anxiety and uncertainty in men undergoing active surveillance (AS). Developing a psychosocial support program for such patients might promote psychosocial well-being and patient engagement. This review aims to identify interventions with the potential to influence the psychosocial burden of prostate cancer patients undergoing AS.

**Methods:**

A scoping review was conducted in accordance with the PRISMA Extension for Scoping Reviews Checklist. A systematic search was conducted in six databases and included publications dating from 2009. All available and eligible evidence was included in this review.

**Results:**

After screening 2824 articles, 12 studies were included in the review: nine quantitative, one qualitative, and two mixed method papers. The relative strength of these studies was limited and the quality of most was moderate.

**Conclusions:**

The described interventions can be categorized into three major themes: information and education, coping and (psycho)social support, and lifestyle. Psychosocial support for men undergoing AS should entail involvement of family and spouse during the decision-making process, tailored information about PCa treatments, risks, benefits, protocols, lifestyle adjustments, and complementary and alternative medicine. Assessment and promotion of effective coping and self-management strategies are recommended. Healthcare providers should actively promote physical activity and nutritional improvements. Physical activity programs may also be helpful in facilitating peer support, which is especially important for men with limited social support. Future research should investigate combining interventions to increase efficacy and optimize supportive care during AS.

**Supplementary Information:**

The online version contains supplementary material available at 10.1007/s00520-022-06830-z.

## Introduction

In 2020, approximately 1.4 million men were diagnosed with prostate cancer (PCa) worldwide [1]. The incidence is still increasing and it is estimated that around 2.3 million men will be diagnosed with PCa annually by 2040 [1]. A large proportion of these men are diagnosed with low-risk or insignificant disease [2], and various studies have suggested that men with insignificant or low-risk PCa (LR-PCa) do not benefit from radical treatment. In this patient population, expectant management is warranted [3]. During expectant management, patients do not undergo active treatment but remain under close surveillance. Expectant management can be subdivided into active surveillance (AS) and watchful waiting (WW) [4].

AS has a curative intent and consists of aggressive observation to detect early disease progression by monitoring several predefined parameters [4]. If disease progression is detected, radical curative treatment is initiated [5]. In contrast, WW is a palliative option that is most often provided to fragile or older patients when curative or invasive treatment is not desirable (e.g., because of limited life expectancy) [4].

AS is a cost-effective treatment option for LR-PCa [6]. Men who choose AS also avoid complications related to radiotherapy or surgery, such as erectile dysfunction and urinary incontinence [7]. As a result, men undergoing AS generally have greater quality-adjusted life-year scores [8]. Despite these advantages, living with untreated cancer and frequent medical examination may cause anxiety and uncertainty in men undergoing AS [9]. The psychosocial burden of AS has been widely discussed in the literature [10]. A recent study suggests that approximately 30% of patients are at risk of developing anxiety during the first year of AS [11]. And approximately 5–10% of men initiate radical treatment based on anxiety without disease progression present [12, 13]. A decrease in psychosocial well-being may influence patients’ adherence to AS and lead them to initiate curative treatment without disease progression [9, 10, 14].

It has been suggested that there is a greater need for psychosocial support among PCa patients undergoing AS than among patients in active treatment groups [15]. Psychosocial support can be effective in reducing anxiety and uncertainty [16], and providing professional psychosocial support during the first year of AS might enhance its appeal and improve adherence [9–11, 17].

This scoping review aims to identify interventions with the potential to alleviate the psychosocial burden experienced by PCa patients undergoing AS. These interventions include coping strategies, information and psychoeducation, symptom management, lifestyle, complementary and alternative medicine (CAM), self-management, mindfulness, and (peer) support [18]. This research may contribute to developing a psychosocial support program for AS patients that increases psychosocial well-being and patient engagement. This review aimed to answer the following research question: *What interventions affect the psychosocial burden experienced by prostate cancer patients undergoing active surveillance*?

## Methods

A scoping review was conducted in accordance with the PRISMA Extension for Scoping Reviews Checklist with the aim of being systematic, transparent, and replicable [19]. No ethical approval was required and no protocol was registered.

In contrast to a systematic review, a scoping review provides a more general overview of evidence regarding a broader subject. Scoping reviews are used to inform healthcare practice and map the available evidence on a relevant topic. Since the aim of this review is to identify evidence about interventions that may alleviate psychosocial burden during AS, a scoping review is a suitable design for comprehensively and broadly exploring research on this topic [20, 21].

### Search strategy

Between August and October 2020, a systematic scope search was conducted in the following databases with the assistance of a medical librarian: Cinahl plus with full text, Cochrane, Embase (OVID), Psychinfo (Ebsco), PubMed, and Web of Science. Database-specific search strategies are described in Appendices 1 and 2.

The search strings for all databases are provided in Appendix 1. The results of each search were exported into Rayyan [22], and the deduplication process described by Bramer [23] was performed (Appendices 3, 4, 5, and 6).

The first reviewer (KD) screened all titles and abstracts for mention of prostate cancer, active management (i.e., active surveillance), and interventions influencing psychosocial burden during AS. Articles were either deemed eligible for full-text screening or excluded. A subset of articles was independently screened by blind reviewers (LM, BL, EC).

Following title and abstract screening, full texts of all remaining articles were obtained and read. A selection was then made, based on the inclusion and exclusion criteria. In accordance with the nature of a scoping review, all study types and research designs were included. This included both original studies and reviews that presented new results or interpretations not described in included individual studies.

A hand search of the reference lists of included studies and a forward reference check was conducted to identify additional eligible articles. When hand searching revealed new titles, the process of screening and application of in and exclusion criteria was repeated.

### Selection criteria

In and exclusion criteria were used to assess eligibility for this review. Articles were considered eligible for inclusion if they meet the following criteria:

- A peer-reviewed full-text article was available (reporting empirical qualitative, quantitative, or mixed method studies and literature reviews)
- Articles were published in English or Dutch (for practical reasons)
- Research involved adult male humans (generalizability)
- Publication date was in or after 2009 (when a clear distinction between AS and WW was introduced in clinical practice) [24, 25]
- Research on interventions with a potential effect on psychosocial burden during AS
- Research in men with LR-PCa (AS is justified and safe in LR-PCa)

Articles were excluded from this review for the following criteria:

- Research (primarily/exclusively) on the influence of race, ethnicity, age, socioeconomic status, or health literacy on psychosocial burden (not influenced by a psychosocial support intervention)
- The research population consisted of patients undergoing WW and not AS, or the article made no distinction between WW and AS (WW is a palliative option)
- Research on the influence or effects of focal therapy, i.e., HIFU/cryotherapy (no expectant management)
- Research on the influence of medication on disease progression, i.e., statins or androgen deprivation therapy (no expectant management)
- Research that compared levels of quality of life, distress, anxiety, or uncertainty between treatment groups (does not investigate the effect of interventions on the psychosocial burden within AS treatment group)
- Research on the decision-making process and considerations before beginning AS

### Data extraction

A data extraction sheet was used to derive all relevant data from qualitative and quantitative studies. Hard copy data extraction sheets are stored by the reviewer (KD).

### Quality appraisal

Quality appraisal tools were used to assess the quality of the included evidence and assess the level of evidence [26–29]. The quality of included studies was ranked as “high,” “moderate,” or “low.” Two reviewers (KD, BL) discussed various cutoff values until consensus was reached. This corresponds with the scores described in Online Resource 1.

## Results

### Search results

**Fig. 1:**
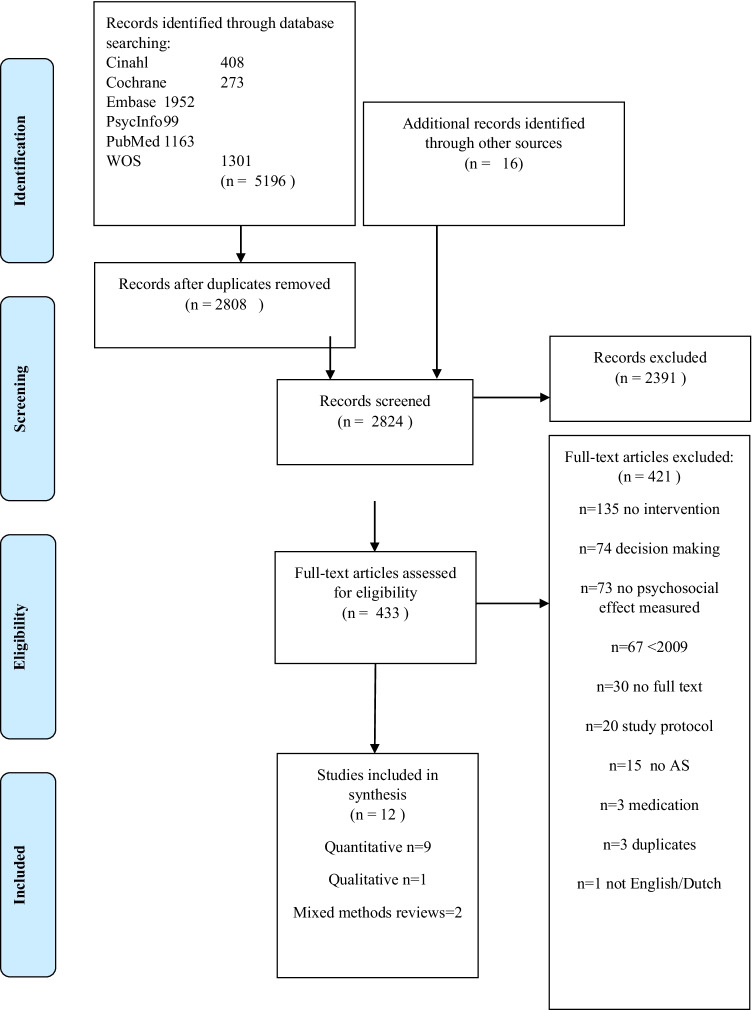
PRISMA flowchart

The literature search was carried out in six scientific databases and yielded 2808 unique citations. A supplementary hand search identified 16 more citations. A flowchart of the search strategy is provided in Fig. 1.

Blind reviewers (LM, BL, EC) independently screened a random subset of 1100 articles. An 82.5% consensus rate on this subset was established. The remaining conflicting results were discussed by the reviewers until consensus was reached.

### Characteristics of the studies

After the full-text screening, 12 studies met all criteria and were included in the review [30–41]. This included nine quantitative [30–33, 35, 39–41], one qualitative [37], and two mixed method [34, 36] papers. All studies involved men with LR-PCa who were under active management. An overview of study characteristics is provided in Table 1.

**Table 1:** Characteristics of included studies [30–41]

Study	Year	Research design	Sample	Duration	Country	Intervention	Outcome/themes	Results/findings
Baba et al	2021	Observational cross-sectional	*N* = 130		Germany	Psychological and social support	Distress, depression, and anxiety	33.3% elevated distress. 16.5% depressive symptoms, 13% anxiety. Psychosocial support increased in distressed patients
Berg et al	2016	Cohort study	*N* = 235	Median 42 months	USA	Diet and food supplements	Adherence	11.5% AS discontinuation
Hedden et al	2017	Pretest posttest	*N* = 71		Canada	Educational session	Anxiety, distress, uncertainty	Reduction in distress (*p* = 0.02)
Kazer et al	2011	Pretest posttest	*N* = 9	5 weeks	USA	5-week internet intervention that entails cognitive reframing, lifestyle, and education	Self-efficacy, uncertainty, QoL	Correlation (*r* = 0.88, *p* = 0.02) between increased QoL and use internet intervention
Kinsella et al	2018	Mixed method review	*N* = 64	n/a	UK	Social support	Adherence	Increased adherence (*p* = 0.001)
Kinsella et al	2019	Quasi-experiment	*N* = 255	5 years	UK	Single educational seminar	Adherence	Increased AS adherence in intervention group (*p* = 0.001)
McIntosh et al	2019	Mixed method systematic review	*N* = 8	n/a	Australia	Support needs	Adherence	Information, psychological and emotional support, social support
Oliffe et al	2009	Qualitative semi-structured interviews	*N* = 25	45–90 min	Canada	Social, peer and spousal support, coping, diet, CAM	Uncertainty	“Living a normal life” coping leads to decreased social support. “Doing something extra” coping leads to increased uptake of lifestyle modifications
Papadopoulos et al	2020	Retrospective cohort study	*N* = 630	2001–2016	Canada	Self-reported physical activity	Quality of life, emotional well-being	High physical activity leads to increased QoL (*p* = 0.002) and increased emotional well-being (*p* = 0.10)
Sumiyoshi et al	2010	Pre-experiment (time series)	*N* = 74	6 months	Japan	Mushroom mycelium extract	Anxiety	Anxiety decreased (*p* = 0.0018 and *p* = 0.0099)
Thomas et al	2014	Double-blind RCT	*N* = 199	6 months	UK	Polyphenol-rich food supplement	Adherence	Increased AS continuation in intervention group (*p* = 0.014)
Victorson et al	2016	Pilot RCT	*N* = 43	12 months	USA	Mindfulness meditation training	Anxiety, uncertainty	Anxiety decreased (*p* = 0.04) and QoL increased (*p* < 0.05) within intervention groups

*n/a* not available.

### Quality appraisal

The results of the quality appraisal are provided in Table 2. None of the 12 studies met all quality criteria, and only three met more than 80% of the quality criteria and were considered to be of high quality [30, 34, 40].

**Table 2:** Quality scores and levels of evidence

Study	Year	Research design	Quality tool	Quality score	Quality appraisal	Level of evidence
Baba et al	2021	Observational cross-sectional	Law et al	11	High	Level VI
Berg et al	2016	Cohort study	Law et al	9	Moderate	Level IV
Hedden et al	2017	Pretest posttest	Law et al	11	Moderate	Level III
Kazer et al	2011	Pretest posttest	Law et al	11	Moderate	Level III
Kinsella et al	2019	Quasi-experiment	Law et al	9	Moderate	Level III
Kinsella et al	2018	Mixed method review	Hong et al	14	High	Level V
McIntosh et al	2019	Mixed method systematic review	Hong et al	13	Moderate	Level V
Oliffe et al	2009	Qualitative semi-structured interviews	Tong et al	20	Moderate	Level VI
Papadopoulos et al	2020	Retrospective cohort study	Law et al	11	Moderate	Level IV
Sumiyoshi et al	2010	Pre-experiment (time series)	Law et al	10	Moderate	Level III
Thomas et al	2014	Double-blind RCT	Law et al	13	High	Level II
Victorson et al	2016	Pilot RCT	Law et al	10	Moderate	Level II

### Themes

Three major intervention themes emerged from this review: information and education, coping and (psycho)social support, and lifestyle. A narrative synthesis of the results from the included studies is provided.

#### Information and education

Five studies investigated the relationship between information, education, and the psychosocial burden of AS [32–36]. These studies confirmed the presence of unmet informational needs among PCa patients eligible for, or currently undergoing, AS.

The systematic review carried out by McIntosh et al. identified unmet informational needs [36]. PCa patients undergoing AS often receive information that is inadequate, confusing, and inconsistent, which causes them distress. They receive inadequate information about PCa, signs of progression, future treatment options, and adjuvant treatment such as diet and exercise.

Ideally, the information should be consistent, unambiguous, concise, and adequately tailored to the individual. This conclusion was supported by the systematic review conducted by Kinsella et al., which identified facilitators and barriers to AS adherence [34]. Their review affirmed the importance of customization and personalization of just-in-time information. It also stressed that information should not only be provided to patients, but education must involve partners and family. Inaccurate risk assessment can cause family and friends to pressure patients to undergo curative treatment.

The importance of educating patients and their partners was affirmed by the results of the quasi-experiment conducted by Hedden et al. [32], which evaluated the effect of an educational intervention on distress and anxiety among men diagnosed with LR-PCa (*n* = 71) and their partners (*n* = 48). The study demonstrated that partners had higher distress levels than patients before attending an educational seminar (*p* = 0.03) about PCa and treatments. That seminar addressed the informational needs identified by McIntosh et al. [36]. Customized and personalized information was also provided during a private meeting between the patient, partner, urologist, and radio-oncologist. Hedden et al. found that attending the seminar and private meeting significantly decreased distress among patients and partners compared to their distress before the seminar (*p* =  < 0.001).

Kinsella et al. assessed the long-term effects of a seminar on AS adherence [35]. They conducted a quasi-experiment to assess the effect of a single 1.5-h educational seminar, after which time was scheduled for questions and peer discussion. During the first year of the study, no one experienced clinical disease progression in the intervention (*n* = 120) or control (*n* = 135) group. However, a significantly smaller proportion of men in the intervention group (11%, *p* = 0.003) dropped out of AS compared to the control group (25%). After 5 years, the intervention group continued to demonstrate increased adherence to AS with a significantly smaller dropout rate among patients with no evidence of disease progression (*p* < 0.001).

Kazer et al. conducted a quasi-experimental study to investigate the effect of an internet-based information and education intervention on older men undergoing AS (aged 66–79, *x̄* = 72) [33]. The intervention was combined with tailored information provided via email by a specialized nurse upon request. Cognitive reframing and self-management strategies were also provided. After subjects completed the intervention, researchers observed an overall trend toward an increase in quality of life (QoL). This study suggests that it is feasible to provide information online.

Successful reassurance and education of the patient and family are key facilitators to AS adherence. Health literacy also plays an important role, and provision and access to relevant and understandable information is a consistent theme. Inaccurate risk and survival perceptions by patients and their families may increase distress and decrease AS uptake and adherence. In contrast, distress is decreased and AS uptake and adherence are increased by the provision of transparent, consistent, and understandable information regarding PCa diagnosis, prognosis, treatment options, active surveillance, monitoring protocols, AS-associated risks, and reliable guidelines on complementary options, diet, and lifestyle changes.

#### Coping and (psycho)social support

Five studies addressed the importance of coping and social support during AS [30, 33, 34, 36, 37]. A cross-sectional study conducted by Baba et al. found that distressed patients undergoing AS have an elevated need for psychosocial support [30]. The systematic review conducted by McIntosh et al. found that distress is caused by unmet emotional, psychological, and social support needs [36]. In particular, men who experience AS negatively have more difficulty dealing with AS. That was less complicated for men who felt that AS was “having a positive impact,” according to Kinsella et al. [34]. They found that such men experience less uncertainty and feel more in control. Baba et al. found something similar in their cross-sectional study of men with PCa (*n* = 130) who were undergoing AS (*n* = 19) [30]. They claimed that a “positive attitude” was helpful to promote coping with AS-associated distress.

Oliffe et al. affirmed the importance of adopting coping strategies [37]. Their qualitative interview study identified two self-management strategies for alleviating AS-induced uncertainty in a sample of 25 men: “living a normal life” and “doing something extra.” The study also assessed the implications of these strategies. Although “living a normal life” seemed to be an effective strategy for avoiding worry and distress, it was associated with a limited support network, avoidance, stoicism, and a resistance to lifestyle changes. Thus, this strategy may decrease AS compliance. In contrast, adopting a “doing something extra” strategy seemed to promote positive lifestyle changes. This strategy is characterized by engagement in self-health and education. Oliffe et al. observed an increased use of CAM in this population. Engagement with their partner, family, and friends seemed to affirm the patients’ commitment to AS and increase adherence.

The finding that social support promotes coping with AS corresponds with the results from other studies in this review. The review conducted by Kinsella et al. demonstrated a strong correlation between AS adherence and AS support groups for men and their families [34]. This was confirmed by Baba et al., whose study indicated that peer support from interpersonal relationships with partners, friends, and family helps patients cope with AS [30]. McIntosh et al. recommend referring men to (online anonymous) support groups during AS [36].

Psychotherapeutic interventions may help men who are unable to cope with AS. Kazer et al. investigated a cognitive reframing behavioral intervention among men (*n* = 9) and found a positive correlation between intervention usage and QoL, especially in the domains “role function related to emotional health” and “social function” (*r* = 0.88, *p* = 0.02) [33].

In summary, men who have unmet support needs during AS are at increased risk of distress. Adopting helpful coping and self-management strategies, such as a positive attitude and a “doing something extra” strategy, may improve patients’ ability to cope with AS. Furthermore, social support promotes coping, AS adherence, and commitment. Social support can be present within personal relationships or facilitated through peer support groups. Professional psychotherapeutic help may be beneficial to men who feel unable to cope with AS.

#### Lifestyle

Eight studies examined the uptake and effects of lifestyle adjustments [30, 31, 33, 34, 37–41]. Most of them focused on diet and food supplements [31, 33, 34, 39, 40]. According to Oliffe et al., engagement in self-health is associated with increased commitment and adherence to AS [37]. The review conducted by Kinsella et al. found that self-management strategies, such as exercise and stress management, help men cope with uncertainty during AS [34]. Baba et al. also stated that physical activity was helpful for men who were dealing with distress [30].

These findings were confirmed by the retrospective cohort study conducted by Papadopoulos et al. [38], which explored the association between self-reported physical activity, QoL, and emotional well-being in a cohort of 630 men. Highly active participants showed significantly higher (*p* = 0.002) QoL scores. The most active group also demonstrated the highest odds of experiencing high emotional well-being (*p* = 0.010).

Kazer et al. investigated the effect of an internet-based intervention that combined various components, including nutritional recommendations and the promotion of physical activity [33]. This incorporated weight control, exercise, limiting meat, and avoiding alcohol and smoking. Their four-component intervention resulted in an overall trend toward improved QoL. After patients completed the intervention period, their QoL returned toward baseline, indicating that the intervention had a temporary and transient effect.

Diet and nutritional recommendations were also investigated in the large longitudinal cohort study conducted by Berg et al. [31]. They examined the effect of a holistic AS approach on adherence in a sample of 235 men. Holistic AS consisted of AS monitoring complemented with strict dietary recommendations. In this study, with a median follow-up of 42 months, only 1.3% of patients discontinued AS because of anxiety or uncertainty. In general, researchers observe a dropout of 5–10% due to anxiety and uncertainty [13].

Thomas et al. conducted a double-blind placebo-controlled randomized trial to evaluate the effect of a polyphenol-rich whole food supplement on AS discontinuation [40]. They found that a statistically significantly lower proportion of men in the food supplement group (*n* = 134) discontinued AS (*p* = 0.014). Their reasons for discontinuing AS were not disclosed.

Only the study by Sumiyoshi et al. assessed the direct effect of a food supplement on psychosocial well-being [39]. They examined the effect of dietary administration of mushroom mycelium extract (AHCC) on anxiety during a quasi-experiment among 74 men treated with expectant management. Significantly lower State-Trait Anxiety Index (STAI) scores were observed in participants with high anxiety levels before the intervention (*p* < 0.01).

Anxiety and uncertainty also significantly decreased as a result of participation in an 8-week mindfulness meditation training, according to Victorson et al. [41]. Results from their pilot randomized controlled trial demonstrated a significant (*p* = 0.04) within-group decrease of anxiety in the intervention group (*n* = 24) between baseline and 6 months after the intervention. Uncertainty decreased between baseline and 12 months (*p* < 0.01), and global mental health increased between baseline and 8 weeks (*p* < 0.05). A between-group analysis indicated that post-traumatic growth was the outcome that significantly increased in the intervention group (*p* = 0.01) compared to the control group (*n* = 19).

Overall, engaging in self-health by making healthy lifestyle adjustments seems to provide men with important self-management strategies during AS, resulting in decreased distress, anxiety, and uncertainty and improved AS adherence. In addition, some lifestyle adjustments seem to directly affect anxiety and uncertainty. For instance, exercise seems to directly benefit emotional well-being, and participation in a mindfulness meditation program decreased anxiety and uncertainty.

## Discussion

This scoping review identified interventions that affect the psychosocial burden experienced by PCa patients undergoing AS. Those interventions can be categorized into three major themes: information and education, coping and psychosocial support, and lifestyle.

In the first category, providing information about PCa, treatments, AS protocols, prognosis, mortality, morbidity, and lifestyle via a website or educational seminar seems to assist in risk assessment and decrease uncertainty [32, 36]. A positive impact on AS adherence is associated with these interventions. The involvement of the patient’s partner and family is critical to the provision of information since they seem to play an important role in reassuring the patient and facilitating AS adherence [32, 36]. In addition, the information should be tailored to the needs and health literacy levels of the patient and their family [34]. Individual meetings or tailored contacts could provide opportunities to assess whether patients have obtained all the required information and whether that information was consistent, understandable, and relevant [33].

Selecting AS should be the result of a thorough consideration of risks, benefits, and coping and self-management strategies. It should not merely be selected to avoid treatment-related complications. Thus, it is recommended that shared decision-making techniques and decision aids be used to support careful deliberation about AS [42–44].

The second category comprised coping and psychosocial support interventions [30, 33–35, 37]. Adopting a positive attitude toward AS seems to decrease distress and uncertainty [30, 34]. Although an avoidant self-management strategy enables patients to temporarily prevent stress, uncertainty, and anxiety associated with AS, this strategy is also associated with a limited support network and a resistance to lifestyle changes [33, 37]. These last two features are negatively associated with AS adherence. Adopting a self-management strategy that emphasizes self-health, self-education, and engagement with family and friends seems to improve AS adherence.

It is recommended to assess the adoption of effective coping and self-management strategies. Healthcare providers can use specially designed questionnaires to identify anxiety, uncertainty, or distress [45–47], and professional psychological support should be offered to men who are unable to cope with AS [36]. Cognitive behavior therapy, especially cognitive reframing, might help to promote a positive attitude [33]. Social support from peers, family, and friends also plays an important role in helping patients deal with the psychosocial burden associated with AS. Some patients may benefit from peer support, especially those who have a limited social support network or who do not wish to discuss their experience with their friends or family [30, 34, 36, 37].

Lifestyle interventions and adjustments comprise the final category [30, 31, 33, 34, 37–41]. Physical activity, exercise, and mindfulness seem to directly affect distress, QoL, and uncertainty [30, 33, 34, 38, 41]. In addition, dietary recommendations and nutritional supplements may positively influence AS adherence [31, 33, 34, 39, 40]. It is most likely that lifestyle adjustments provide a self-management strategy that helps patients cope with uncertainty during AS [37]. Engaging in self-health practices seems to promote a sense of control over one’s disease that directly and indirectly affects the psychosocial burden experienced during AS. Patients and their partners actively seek information about dietary and exercise interventions [48]. Providing such recommendations may lead some men to make positive lifestyle changes. However, others may need professional support and guidance (e.g., from a nutritional or physical therapist) to facilitate lifestyle improvements. Motivational interviewing might improve the uptake of healthy lifestyle changes [49, 50].

### Study strengths and limitations

The results of this review are based on a comprehensive and systematic search of multiple databases. The search and selection processes have been described in detail. In addition, the quality of the available evidence was assessed and a quality appraisal is provided. Despite the strengths of this scoping review, it has some limitations. One is that it was conducted by a single researcher. However, three independent reviewers were involved in selecting the articles to be reviewed. The methodology and process also were extensively discussed within the supervisory team. This decreases the risk of measurement bias.

A second limitation is the quality of the included evidence. None of the included studies met all quality criteria. In addition, the level of evidence of the included studies was predominantly low to medium. Of the two high-level studies included, one was a pilot study that therefore had a small sample size. This may have influenced the validity of the results. However, this review imposed no restrictions on research designs and thus included all available and eligible evidence in this review.

In addition, this review identified interventions that mostly had a short-term, positive effect [33], and most of the studies investigated stand-alone interventions. Therefore, it is unclear whether combining interventions can have a complementary effect and whether a combination of interventions would generate longer-lasting or improved results (e.g., a combination of interventions that have a transitory effect with interventions that demonstrate long-term results) [35].

## Conclusion

In conclusion, various psychosocial interventions and lifestyle adjustments seem to affect the psychosocial burden experienced by prostate cancer patients undergoing active surveillance. Based on the findings from this review, various interventions appear to decrease the psychosocial burden during AS. Firstly, this review emphasizes the importance of transparent, understandable, relevant, and reliable information about PCa, treatments, AS risks, benefits, and protocols. In addition, information on lifestyle adjustments and CAM should be provided. Secondly, families and partners should receive adequate information and be involved in the decision-making process. Moreover, assessing the coping and self-management strategies used by men undergoing AS can identify those who find it difficult to handle. Men who are struggling with AS or have limited coping skills should be encouraged to adopt helpful coping strategies, such as a positive attitude and health-promoting behavior. Professional psychological support should be offered to men unable to deal with AS. Furthermore, healthcare providers (e.g., urologists, nurse specialists, nurses) should actively promote physical activity and nutritional improvements by providing exercise and dietary recommendations or organizing lifestyle support programs. Physical activity programs for men undergoing AS may also be helpful in facilitating peer support.

In regard to the limitations of this review, the included number of studies, and their quality, it is recommended that future research investigates the feasibility and efficacy of combining various interventions that may potentially decrease psychosocial burden and optimize supportive care during AS.

## Supplementary Information

Below is the link to the electronic supplementary material.

Supplementary file1 (DOCX 15 KB)

Supplementary file2 (DOCX 27 KB)

## Data Availability

The data that support the findings of this study are available on request from the corresponding author, KD.
