# Supplementary material for: What interventions affect the psychosocial burden experienced by prostate cancer patients undergoing active surveillance? A scoping review
Source: Support Care Cancer. 2022 Jan 26;30(6):4699–709. doi: 10.1007/s00520-022-06830-z (PMC9046366; doi:10.1007/s00520-022-06830-z)
Supplement: Supplementary file 1 — Supplementary file1 (DOCX 15 KB) [file 520_2022_6830_MOESM1_ESM.docx]

**REVIEW TITLE**

What interventions affect the psychosocial burden experienced by prostate cancer patients undergoing active surveillance? A scoping review

**AUTHOR INFORMATION**

Kim Donachie | Erik Cornel | Thomas Pelgrim| Leslie Michielsen | Bart Langenveld | Marian Adriaansen | Esther Bakker | Lilian Lechner

**CORRESPONDING AUTHOR**

K.M. Donachie, MSc, RN

ORCID ID: 0000-0002-7367-2348

HAN University of Applied Sciences, Academy of Health,

P.O. Box 6960, 6503 GL Nijmegen.

Email: kim.donachie@han.nl

**Online Resource 1. Quality scoring**

| Quality Appraisal Tool | High | Moderate | Low |
| --- | --- | --- | --- |
| Law, et al (McMaster) | >12 | >9 | <8 |
| Tong, et al (COREQ) | >26 | >19 | <18 |
| Hong, et al (MMAT) | >14 | >10 | <9 |

**Online Resource 2. Levels of evidence**

| Level of evidence | Description |
| --- | --- |
| Level I | Systematic review & meta-analysis of randomized controlled trials. |
| Level II | >1 Randomized controlled trials |
| Level III | Quasi-experiment |
| Level IV | Case-control or cohort study |
| Level V | Systematic review of descriptive & qualitative studies |
| Level VI | Single descriptive or qualitative study |
| Level VII | Expert opinion |
