# Supplementary material for: What interventions affect the psychosocial burden experienced by prostate cancer patients undergoing active surveillance? A scoping review
Source: Support Care Cancer. 2022 Jan 26;30(6):4699–709. doi: 10.1007/s00520-022-06830-z (PMC9046366; doi:10.1007/s00520-022-06830-z)
Supplement: Supplementary file 2 — Supplementary file2 (DOCX 27 KB) [file 520_2022_6830_MOESM2_ESM.docx]

**Appendix 1: Cinahl versie 1 2-10-2020**

S1 MH "Prostatic Neoplasms" OR TI ((Prostat* N1 (neoplas* OR cancer* OR carcinoma* OR adenocarcinoma* OR tumor* OR tumour* OR malignancy)) OR PCa OR “LR-PCa”) OR AB ((Prostat* N1 (neoplas* OR cancer* OR carcinoma* OR adenocarcinoma* OR tumor* OR tumour* OR malignancy)) OR PCa OR “LR-PCa”) OR SU ((Prostat* N1 (neoplas* OR cancer* OR carcinoma* OR adenocarcinoma* OR tumor* OR tumour* OR malignancy)) OR PCa OR “LR-PCa”)

<https://blocks.bmi-online.nl/catalog/285>

S2 TI ((watchful N1 waiting) OR (active N1 surveillance) OR untreated OR expectant OR expectative OR (active N1 monitor*) OR ((delayed OR deferred) N1 (treat* OR manag*)) OR expectant OR expectative) OR AB ((watchful N1 waiting) OR (active N1 surveillance) OR untreated OR expectant OR expectative OR (active N1 monitor*) OR ((delayed OR deferred) N1 (treat* OR manag*)) OR expectant OR expectative) OR SU ((watchful N1 waiting) OR (active N1 surveillance) OR untreated OR expectant OR expectative OR (active N1 monitor*) OR ((delayed OR deferred) N1 (treat* OR manag*)) OR expectant OR expectative)

S3 MH "Emotions+" OR MH "Anxiety Disorders" OR TI (anxiety* OR anxious OR panic* OR (panic N1 attack*) OR fear* OR scared* OR afraid* OR terrify OR terrified* OR fright* OR dread* OR despair) OR AB (anxiety* OR anxious OR panic* OR (panic N1 attack*) OR fear* OR scared* OR afraid* OR terrify OR terrified* OR fright* OR dread* OR despair) OR SU (anxiety* OR anxious OR panic* OR (panic N1 attack*) OR fear* OR scared* OR afraid* OR terrify OR terrified* OR fright* OR dread* OR despair)

S4 MH "Uncertainty" OR TI (uncertainty OR insecure OR Worrie* OR worry OR apprehension OR nervousness OR jitter* OR uneas* OR restlessness* OR concern*) OR AB (uncertainty OR insecure OR Worrie* OR worry OR apprehension OR nervousness OR jitter* OR uneas* OR restlessness* OR concern*) OR SU (uncertainty OR insecure OR Worrie* OR worry OR apprehension OR nervousness OR jitter* OR uneas* OR restlessness* OR concern*)

S5 MH "Quality of Life+" OR TI ((quality N1 life) OR QoL OR “HR-QoL”) OR SU ((quality N1 life) OR QoL OR “HR-QoL”)

S6 MH "Mental Health" OR MH "Stress, Psychological" OR MH "Psychological Distress" OR TI (((mental OR Emotional) N3 (wellbeing OR “well-being” OR suffering)) OR distress OR stress OR Mental OR (negative N1 (feeling* OR emotion* OR thought*)) OR suffer* OR burden OR Psychological) OR AB (((mental OR Emotional) N3 (wellbeing OR “well-being” OR suffering)) OR distress OR stress OR Mental OR (negative N1 (feeling* OR emotion* OR thought*)) OR suffer* OR burden OR Psychological) OR SU (((mental OR Emotional) N3 (wellbeing OR “well-being” OR suffering)) OR distress OR stress OR Mental OR (negative N1 (feeling* OR emotion* OR thought*)) OR suffer* OR burden OR Psychological)

S7 MH "Adaptation, Psychological" OR MH "Coping" OR MH "Self-Management" OR TI ((psychological N1 adapt*) OR coping OR cope OR (Adaptive N1 (Behavior* OR Behaviour*)) OR adaption OR (Self N1 management) OR Selfmanagement OR (emotional N1 respons*)) OR AB ((psychological N1 adapt*) OR coping OR cope OR (Adaptive N1 (Behavior* OR Behaviour*)) OR adaption OR (Self N1 management) OR Selfmanagement OR (emotional N1 respons*)) OR SU ((psychological N1 adapt*) OR coping OR cope OR (Adaptive N1 (Behavior* OR Behaviour*)) OR adaption OR (Self N1 management) OR Selfmanagement OR (emotional N1 respons*))

S8 MH "Depression" OR TI (depress* OR sadness OR somber OR sorrow OR grief OR grieving OR unhapp* OR desolat*) OR AB (depress* OR sadness OR somber OR sorrow OR grief OR grieving OR unhapp* OR desolat*) OR SU (depress* OR sadness OR somber OR sorrow OR grief OR grieving OR unhapp* OR desolat*)

S9 MH "Sexuality+" OR MH "Masculinity" OR TI (mascul* OR intim* OR selfesteem OR (self N1 esteem) OR (self N1 image) OR (self N1 confiden*) OR selfconfidence OR sexual OR confiden*) OR AB (mascul* OR intim* OR selfesteem OR (self N1 esteem) OR (self N1 image) OR (self N1 confiden*) OR SU (mascul* OR intim* OR selfesteem OR (self N1 esteem) OR (self N1 image) OR (self N1 confiden*) OR selfconfidence)

**S10 MH "Alternative Therapies+" OR MH "Exercise+" OR MH "Peer Group" OR MH "Support Groups" OR MH "Life Style+" OR MH "Diet Therapy+" OR MH "Diet+" OR MH "Spouses" OR MH "Cognitive Therapy+" OR MH "Health Education" OR MH "Support, Psychosocial+" OR MH "Health Services Needs and Demand" OR TI ((mental N1 support) OR food OR nutrition* OR relax* OR (physical N1 activit*) OR exercise* OR sport* OR fitness OR CAM OR ((complementary OR alternative) N1 therap*) OR aroma* OR hypno* OR massag* OR acupressure OR acupuncture OR mindful* OR meditation* OR workout* OR lifestyl* OR (life N1 style) OR diet OR (Cognit* N1 (Therap* OR Psychotherap*) OR (behavio* N1 intervention*) OR counsel*)) OR AB ((mental N1 support) OR food OR nutrition* OR relax* OR (physical N1 activit*) OR exercise* OR sport* OR fitness OR CAM OR ((complementary OR alternative) N1 therap*) OR aroma* OR hypno* OR massag* OR acupressure OR acupuncture OR mindful* OR meditation* OR workout* OR lifestyl* OR (life N1 style) OR diet OR (Cognit* N1 (Therap* OR Psychotherap*) OR (behavio* N1 intervention*)) OR SU ((mental N1 support) OR food OR nutrition* OR relax* OR (physical N1 activit*) OR exercise* OR sport* OR fitness OR CAM OR ((complementary OR alternative) N1 therap*) OR aroma* OR hypno* OR massag* OR acupressure OR acupuncture OR mindful* OR meditation* OR workout* OR lifestyl* OR (life N1 style) OR diet OR (Cognit* N1 (Therap* OR Psychotherap*) OR (behavio* N1 intervention*))**

S11 S3 OR S4 OR S5 OR S6 OR S7 OR S8 OR S9 OR S10

S12 S1 AND S2 AND S11

**Appendix 2: Cochrane versie 1 28-10-2020**

#1 ((Prostat* NEAR/1 (neoplas* OR cancer* OR carcinoma* OR adenocarcinoma* OR tumor* OR tumour* OR malignancy)) OR PCa OR "LR-PCa"):ti,ab,kw

<https://blocks.bmi-online.nl/catalog/285>

#2 ((watchful NEAR/1 waiting) OR (active NEAR/1 surveillance) OR untreated OR expectant OR expectative OR (active NEAR/1 monitor*) OR ((delayed OR deferred) NEAR/1 (treat* OR manag*)) OR expectant OR expectative):ti,ab,kw

#3 (anxiety* OR anxious OR panic* OR (panic NEAR/1 attack*) OR fear* OR scared* OR afraid* OR terrify OR terrified* OR fright* OR dread* OR despair):ti,ab,kw

#4 (uncertainty OR insecure OR Worrie* OR worry OR apprehension OR nervousness OR jitter* OR uneas* OR restlessness* OR concern*):ti,ab,kw

#5 ((quality NEAR/1 life) OR QoL OR "HR-QoL"):ti,kw

#6 (((mental OR Emotional) NEAR/3 (wellbeing OR "well-being" OR suffering)) OR distress OR stress OR Mental OR (negative NEAR/1 (feeling* OR emotion* OR thought*)) OR suffer* OR burden OR Psychological):ti,ab,kw

#7 ((psychological NEAR/1 adapt*) OR coping OR cope OR (Adaptive NEAR/1 (Behavior* OR Behaviour*)) OR adaption OR (Self NEAR/1 management) OR Selfmanagement OR (emotional NEAR/1 respons*)):ti,ab,kw

#8 (depress* OR sadness OR somber OR sorrow OR grief OR grieving OR unhapp* OR desolat*):ti,ab,kw

#9 (mascul* OR intim* OR selfesteem OR (self NEAR/1 esteem) OR (self NEAR/1 image) OR (self NEAR/1 confiden*) OR selfconfidence OR confiden*):ti,ab,kw OR sexual:ti

**#10 ((mental NEAR/1 support) OR food OR nutrition* OR relax* OR (physical NEAR/1 activit*) OR exercise* OR sport* OR fitness OR CAM OR ((complementary OR alternative) NEAR/1 therap*) OR aroma* OR hypno* OR massag* OR acupressure OR acupuncture OR mindful* OR meditation* OR workout* OR lifestyl* OR (life NEAR/1 style) OR diet OR (Cognit* NEAR/1 (Therap* OR Psychotherap*) OR (behavio* NEAR/1 intervention*) OR counsel*))**:ti,ab,kw

#11 #3 OR #4 OR #5 OR #6 OR #7 OR #8 OR #9 OR #10

#12 #1 AND #2 AND #11

**Appendix 3: Embase versie 1 9-10-2020**

1 exp prostate tumor/ OR ((Prostat* ADJ1 (neoplas* OR cancer* OR carcinoma* OR adenocarcinoma* OR tumor* OR tumour* OR malignancy)) OR PCa OR "LR-PCa").ti,ab,kw.

<https://blocks.bmi-online.nl/catalog/285>

2 watchful waiting/ OR ((watchful ADJ1 waiting) OR (active ADJ1 surveillance) OR untreated OR expectant OR expectative OR (active ADJ1 monitor*) OR ((delayed OR deferred) ADJ1 (treat* OR manag*)) OR expectant OR expectative).ti,ab,kw.

3 emotion/ or exp affect/ or exp anger/ or emotion regulation/ or exp fear/ or frustration/ or exp grief/ or happiness/ or helplessness/ or hopelessness/ or mental irritation/ or mood/ or mood change/ or patient worry/ OR exp anxiety/ OR exp anxiety disorder/ OR (anxiety* OR anxious OR panic* OR (panic ADJ1 attack*) OR fear* OR scared* OR afraid* OR terrify OR terrified* OR fright* OR dread* OR despair).ti,ab,kw.

4 uncertainty/ OR ambiguity/ OR exp nervousness/ OR (uncertainty OR insecure OR Worrie* OR worry OR apprehension OR nervousness OR jitter* OR uneas* OR restlessness* OR concern*).ti,ab,kw.

5 "quality of life"/ OR ((quality ADJ1 life) OR QoL OR "HR-QoL").ti,kw.

6 exp mental health/ OR mental stress/ OR psychological balance/ OR psychological resilience/ OR (((mental OR Emotional) ADJ3 (wellbeing OR "well-being" OR suffering)) OR distress OR stress OR Mental OR (negative ADJ1 (feeling* OR emotion* OR thought*)) OR suffer* OR burden OR Psychological).ti,ab,kw.

resilience

7 exp self care/ OR adaptive behavior/ OR exp coping behavior/ OR self monitoring/ OR ((psychological ADJ1 adapt*) OR coping OR cope OR (Adaptive ADJ1 (Behavior* OR Behaviour*)) OR adaption OR (Self ADJ1 management) OR Selfmanagement OR (emotional ADJ1 respons*)).ti,ab,kw.

8 exp depression/ OR sadness/ OR (depress* OR sadness OR somber OR sorrow OR grief OR grieving OR unhapp* OR desolat*).ti,ab,kw.

9 sexuality/ or libido/ or sexual health/ OR sexual satisfaction/ OR masculinity/ OR (mascul* OR intim* OR selfesteem OR (self ADJ1 esteem) OR (self ADJ1 image) OR (self ADJ1 confiden*) OR selfconfidence OR confiden*).ti,ab,kw. OR sexual.ti

**10** life satisfaction/ OR exp lifestyle/ OR "lifestyle and related phenomena"/ or lifestyle modification/ OR exp wellbeing/ OR alternative medicine/ OR exp acupuncture/ OR exp exercise/ OR exp peer group/ OR exp meditation/ OR exp diet therapy/ OR exp diet/ OR exp spouse/ OR exp cognitive behavioral therapy/ OR health education/ or exp health promotion/ or patient education/ or psychoeducation/ or psychosocial care/ OR massage/ OR Hypericum perforatum/ OR herb/ OR peer counseling/ OR exp yoga/ OR physical activity/ or cycling/ or jogging/ or running/ or swimming/ or walking/ or support group/ OR exp social network/ OR social support/ OR exp cognitive therapy/ OR health literacy/ OR **((mental ADJ1 support) OR food OR nutrition* OR relax* OR (physical ADJ1 activit*) OR exercise* OR sport* OR fitness OR CAM OR ((complementary OR alternative) ADJ1 therap*) OR aroma* OR hypno* OR massag* OR acupressure OR acupuncture OR mindful* OR meditation* OR workout* OR lifestyl* OR (life ADJ1 style) OR diet OR (Cognit* ADJ1 (Therap* OR Psychotherap*) OR (behavio* ADJ1 intervention*) OR counsel*))**.ti,ab,kw.

11 3 OR 4 OR 5 OR 6 OR 7 OR 8 OR 9 OR 10

12 1 AND 2 AND 11

13 limit 12 to conference abstract status

14 12 NOT 13

**Appendix 4: PsycInfo versie 1 9-10-2020**

S1 TI ((Prostat* N1 (neoplas* OR cancer* OR carcinoma* OR adenocarcinoma* OR tumor* OR tumour* OR malignancy)) OR PCa OR “LR-PCa”) OR AB ((Prostat* N1 (neoplas* OR cancer* OR carcinoma* OR adenocarcinoma* OR tumor* OR tumour* OR malignancy)) OR PCa OR “LR-PCa”) OR SU ((Prostat* N1 (neoplas* OR cancer* OR carcinoma* OR adenocarcinoma* OR tumor* OR tumour* OR malignancy)) OR PCa OR “LR-PCa”)

<https://blocks.bmi-online.nl/catalog/285>

S2 TI ((watchful N1 waiting) OR (active N1 surveillance) OR untreated OR expectant OR expectative OR (active N1 monitor*) OR ((delayed OR deferred) N1 (treat* OR manag*)) OR expectant OR expectative) OR AB ((watchful N1 waiting) OR (active N1 surveillance) OR untreated OR expectant OR expectative OR (active N1 monitor*) OR ((delayed OR deferred) N1 (treat* OR manag*)) OR expectant OR expectative) OR SU ((watchful N1 waiting) OR (active N1 surveillance) OR untreated OR expectant OR expectative OR (active N1 monitor*) OR ((delayed OR deferred) N1 (treat* OR manag*)) OR expectant OR expectative)

S3 DE "Emotions" OR DE "Contempt" OR DE "Desire" OR DE "Emotional Content" OR DE "Emotional Disturbances" OR DE "Emotional Health" OR DE "Emotional Processing" OR DE "Emotional Regulation" OR DE "Emotional States" OR DE "Emotional Style" OR DE "Expressed Emotion" OR DE "Forgiveness" OR DE "Negative Emotions" OR DE "Positive Emotions" OR DE "Emotion Focused Therapy" OR DE "Emotional Adjustment" OR DE "Emotional Control" OR DE "Identity Crisis" OR DE "Emotional Responses" OR DE "Affective Valence" OR DE "Conditioned Emotional Responses" OR DE "Self-Knowledge" OR DE "Anxiety Disorders" OR DE "Anxiety" OR DE "Anxiety Management" OR DE "Fear" OR DE "Panic" OR DE "Panic Attack" OR TI (anxiety* OR anxious OR panic* OR (panic N1 attack*) OR fear* OR scared* OR afraid* OR terrify OR terrified* OR fright* OR dread* OR despair) OR AB (anxiety* OR anxious OR panic* OR (panic N1 attack*) OR fear* OR scared* OR afraid* OR terrify OR terrified* OR fright* OR dread* OR despair) OR SU (anxiety* OR anxious OR panic* OR (panic N1 attack*) OR fear* OR scared* OR afraid* OR terrify OR terrified* OR fright* OR dread* OR despair)

S4 DE "Uncertainty" OR DE "Ambiguity (Non-Stimulus)" OR DE "Doubt" OR DE "Intolerance of Uncertainty" OR DE "Emotional Security" OR DE "Nervousness" OR TI (uncertainty OR insecure OR Worrie* OR worry OR apprehension OR nervousness OR jitter* OR uneas* OR restlessness* OR concern*) OR AB (uncertainty OR insecure OR Worrie* OR worry OR apprehension OR nervousness OR jitter* OR uneas* OR restlessness* OR concern*) OR SU (uncertainty OR insecure OR Worrie* OR worry OR apprehension OR nervousness OR jitter* OR uneas* OR restlessness* OR concern*)

S5 DE "Quality of Life" OR DE "Health Related Quality of Life" OR DE "Quality of Work Life" OR TI ((quality N1 life) OR QoL OR “HR-QoL”) OR SU ((quality N1 life) OR QoL OR “HR-QoL”)

S6 DE "Mental Health" OR DE "Mental Status" OR DE "Mental Health and Illness Assessment" OR DE "Mental Health Programs" OR DE "Social Health" OR DE "Psychological Stress" OR DE "Stress" OR DE "Psychological Endurance" OR DE "Resilience (Psychological)" OR DE "Stress and Coping Measures" OR DE "Distress" OR TI (((mental OR Emotional) N3 (wellbeing OR “well-being” OR suffering)) OR distress OR stress OR Mental OR (negative N1 (feeling* OR emotion* OR thought*)) OR suffer* OR burden OR Psychological) OR AB (((mental OR Emotional) N3 (wellbeing OR “well-being” OR suffering)) OR distress OR stress OR Mental OR (negative N1 (feeling* OR emotion* OR thought*)) OR suffer* OR burden OR Psychological) OR SU (((mental OR Emotional) N3 (wellbeing OR “well-being” OR suffering)) OR distress OR stress OR Mental OR (negative N1 (feeling* OR emotion* OR thought*)) OR suffer* OR burden OR Psychological)

S7 DE "Self-Care" OR DE "Adaptive Behavior" OR DE "Coping Style" OR DE "Coping Behavior" OR DE "Self-Management" OR DE "Social Emotional Learning" OR DE "Self-Help Techniques" OR DE "Self-Monitoring" OR DE "Self-Regulation" OR TI ((psychological N1 adapt*) OR coping OR cope OR (Adaptive N1 (Behavior* OR Behaviour*)) OR adaption OR (Self N1 management) OR Selfmanagement OR (emotional N1 respons*)) OR AB ((psychological N1 adapt*) OR coping OR cope OR (Adaptive N1 (Behavior* OR Behaviour*)) OR adaption OR (Self N1 management) OR Selfmanagement OR (emotional N1 respons*)) OR SU ((psychological N1 adapt*) OR coping OR cope OR (Adaptive N1 (Behavior* OR Behaviour*)) OR adaption OR (Self N1 management) OR Selfmanagement OR (emotional N1 respons*))

S8 DE "Depression (Emotion)" OR DE "Internalizing Symptoms" OR DE "Major Depression" OR DE "Sadness" OR TI (depress* OR sadness OR somber OR sorrow OR grief OR grieving OR unhapp* OR desolat*) OR AB (depress* OR sadness OR somber OR sorrow OR grief OR grieving OR unhapp* OR desolat*) OR SU (depress* OR sadness OR somber OR sorrow OR grief OR grieving OR unhapp* OR desolat*)

S9 DE "Emotional Inferiority" OR DE "Sexuality" OR DE "Sexual Health" OR DE "Sexual Satisfaction" OR DE "Masculinity" OR DE "Psychology of Men" OR TI (mascul* OR intim* OR selfesteem OR (self N1 esteem) OR (self N1 image) OR (self N1 confiden*) OR selfconfidence OR sexual OR confiden*) OR AB (mascul* OR intim* OR selfesteem OR (self N1 esteem) OR (self N1 image) OR (self N1 confiden*) OR SU (mascul* OR intim* OR selfesteem OR (self N1 esteem) OR (self N1 image) OR (self N1 confiden*) OR selfconfidence)

**S10 DE "Life Satisfaction" OR DE "Lifestyle" OR DE "Lifestyle Changes" OR DE "Well Being" OR DE "Alternative Medicine" OR DE "Acupuncture" OR DE "Aromatherapy" OR DE "Dietary Supplements" OR DE "Antioxidants" OR DE "Vitamins" OR DE "Massage" OR DE "Medicinal Herbs and Plants" OR DE "Hypericum Perforatum" OR DE "Meditation" OR DE "Mind Body Therapy" OR DE "Exercise" OR DE "Physical Activity" OR DE "Aerobic Exercise" OR DE "Yoga" OR DE "Health Behavior" OR DE "Physical Fitness" OR DE "Peer Counseling" OR DE "Peers" OR DE "Support Groups" OR DE "Group Counseling" OR DE "Social Networks" OR DE "Social Support" OR DE "Online Social Networks" OR DE "Diets" OR DE "Spouses" OR DE "Cognitive Therapy" OR DE "Cognitive Behavior Therapy" OR DE "Acceptance and Commitment Therapy" OR DE "Cognitive Processing Therapy" OR DE "Prolonged Exposure Therapy" OR DE "Rational Emotive Behavior Therapy" OR DE "Health Education" OR DE "Client Education" OR DE "Health Knowledge" OR DE "Health Literacy" OR DE "Mental Health Literacy" OR DE "Psychoeducation" OR DE "Psychosocial Readjustment OR DE "Health Service Needs" OR DE "Relaxation" OR DE "Relaxation Therapy" OR TI ((mental N1 support) OR food OR nutrition* OR relax* OR (physical N1 activit*) OR exercise* OR sport* OR fitness OR CAM OR ((complementary OR alternative) N1 therap*) OR aroma* OR hypno* OR massag* OR acupressure OR acupuncture OR mindful* OR meditation* OR workout* OR lifestyl* OR (life N1 style) OR diet OR (Cognit* N1 (Therap* OR Psychotherap*) OR (behavio* N1 intervention*) OR counsel*)) OR AB ((mental N1 support) OR food OR nutrition* OR relax* OR (physical N1 activit*) OR exercise* OR sport* OR fitness OR CAM OR ((complementary OR alternative) N1 therap*) OR aroma* OR hypno* OR massag* OR acupressure OR acupuncture OR mindful* OR meditation* OR workout* OR lifestyl* OR (life N1 style) OR diet OR (Cognit* N1 (Therap* OR Psychotherap*) OR (behavio* N1 intervention*)) OR SU ((mental N1 support) OR food OR nutrition* OR relax* OR (physical N1 activit*) OR exercise* OR sport* OR fitness OR CAM OR ((complementary OR alternative) N1 therap*) OR aroma* OR hypno* OR massag* OR acupressure OR acupuncture OR mindful* OR meditation* OR workout* OR lifestyl* OR (life N1 style) OR diet OR (Cognit* N1 (Therap* OR Psychotherap*) OR (behavio* N1 intervention*))**

S11 S3 OR S4 OR S5 OR S6 OR S7 OR S8 OR S9 OR S10

S12 S1 AND S2 AND S11

**Appendix 5:PubMed versie 5 2-10-2020**

#1 "Prostatic Neoplasms"[Mesh] OR prostatic neoplas*[tiab] OR prostate neoplas*[tiab] OR prostatic cancer*[tiab] OR prostate cancer*[tiab] OR prostatic carcinoma*[tiab] OR prostate carcinoma*[tiab] OR prostatic adenocarcinoma*[tiab] OR prostate adenocarcinoma*[tiab] OR prostatic tumor*[tiab] OR prostate tumor*[tiab] OR prostatic tumour*[tiab] OR prostate tumour*[tiab] OR prostate malignancy[tiab] OR PCa[tiab] OR LR-PCa[tiab]

<https://blocks.bmi-online.nl/catalog/285>

#2 "Watchful Waiting"[Mesh] OR watchful waiting[tiab] OR active surveillance[tiab] OR untreated[tiab] OR expectant[tiab] OR expectative[tiab] OR active monitor*[tiab] OR deferred treat*[tiab] OR deferred manag*[tiab] OR delayed treat*[tiab] OR delayed manag*[tiab] OR expectant[tiab] OR expectative[tiab]

#3 "Emotions"[Mesh] OR "Anxiety"[Mesh] OR "Anxiety Disorders"[Mesh] OR "Fear"[Mesh] OR anxiety*[tiab] OR anxious[tiab] OR panic*[tiab] or panic attack*[tiab] OR fear*[tiab] OR scared*[tiab] OR afraid*[tiab] OR terrify[tiab] OR terrified*[tiab] OR fright*[tiab] OR dread*[tiab] OR despair[tiab]

#4 "Uncertainty"[Mesh] OR uncertainty[tiab] OR insecure[tiab] OR Worrie*[tiab] OR worry[tiab] OR apprehension[tiab] OR nervousness[tiab] OR jitter*[tiab] OR uneas*[tiab] OR restlessness*[tiab] OR concern*[tiab]

#5 "Quality of Life"[Mesh] OR quality of life[ti] OR QoL[ti] OR HR-QoL[ti]

#6 "Stress, Psychological"[Mesh] OR "Psychological Distress"[Mesh] OR emotional wellbeing[tiab] OR emotional well-being[tiab] OR mental wellbeing[tiab] OR mental well-being[tiab] OR distress[tiab] OR stress[tiab] OR mental suffering[tiab] OR negative feeling* [tiab] OR negative emotion*[tiab] OR negative thought*[tiab] OR suffer*[tiab] OR burden[tiab]

#7 "Adaptation, Psychological"[Mesh] **"Mental Health"[Mesh]** OR psychological adapt*[tiab] OR coping[tiab] OR cope[tiab] OR Adaptive Behavior*[tiab] OR Adaptive Behaviour*[tiab] OR adaption[tiab] OR "Self-Management"[Mesh] OR Self-management[tiab] OR Selfmanagement[tiab] OR emotional respons*[tiab] OR Psychological[tiab] OR mental[tiab]

#8 "Depression"[Mesh] OR depress*[tiab] OR sadness[tiab] OR somber[tiab] OR sorrow[tiab] OR grief[tiab] OR grieving[tiab] OR "Sadness"[Mesh] OR unhapp*[tiab] OR desolat*[tiab]

#9 mascul*[tiab] OR "Sexuality"[Mesh] OR sexual*[ti] OR "Masculinity"[Mesh] OR intim*[tiab] OR selfesteem[tiab] OR self esteem[tiab] OR self image[tiab] OR self confidence[tiab] OR confidence[ti] OR selfconfidence[tiab] OR self-confidence[tiab] OR self confidence[tiab]

**#10 "Complementary Therapies"[Mesh] OR "Exercise"[Mesh] OR "Peer Group"[Mesh] OR "Life Style"[Mesh] OR "Healthy Lifestyle"[Mesh] OR "Meditation"[Mesh] OR "Mind-Body Therapies"[Mesh] OR "Nutrition Therapy"[Mesh] OR "Diet"[Mesh] OR "Spouses"[Mesh] OR "Cognitive Behavioral Therapy"[Mesh] OR "Health Education"[Mesh] OR "Psychosocial Support Systems"[Mesh] OR "Cognitive Behavioral Therapy"[Mesh] OR** "Health Services Needs and Demand"[Mesh] **OR counsel*[ti] OR mental support[tiab] OR food[tiab] OR nutrition*[tiab] OR relax*[tiab] OR physical activit*[tiab] OR exercise*[tiab] OR sport*[tiab] OR fitness[tiab] OR CAM[tiab] OR complementary therap*[tiab] OR alternative therap*[tiab] OR aroma*[tiab] OR hypno*[tiab] OR massag*[tiab] OR acupressure[tiab] OR acupuncture[tiab] OR mindful*[tiab] OR meditation*[tiab] OR workout*[tiab] OR lifestyl*[tiab] OR diet[tiab] OR Cognitive Behavioral Therap*[tiab] OR Cognitive Behavior Therap*[tiab] OR Cognitive Psychotherap*[tiab] OR Cognition Therap*[tiab] OR behavioral intervention*[tiab] OR behavior intervention*[tiab] OR behavioural intervention*[tiab] OR behaviour intervention*[tiab]**

#11 #3 OR #4 OR #5 OR #6 OR #7 OR #8 OR #9 OR #10

#12 #1 AND #2 AND #11

**Appendix 6: De-duplication process**

**Searchresults:**

Cinahl 408

Cochrane 273

Embase 1952

PsycInfo 99

PubMed 1163

WOS 1301

**Total 5196**

**De-duplication:**

Round 1 4277

Round 2 3905

Round 3 3569

Round 4 3522

Round 5 3489

Round 6 2808

**Total 2808**
